# Higher amounts of habitual physical activity changes the relationship between hot flashes and subclinical cardiovascular disease risk

**DOI:** 10.14814/phy2.70248

**Published:** 2025-02-13

**Authors:** Sarah Witkowski, Tint Tha Ra Wun, JoSophia Brunzelle, Sara Buszkiewicz, Lorna Murphy, Randi L. Garcia, Lynnette Leidy Sievert

**Affiliations:** ^1^ Department of Exercise & Sport Studies Smith College Northampton Massachusetts USA; ^2^ Department of Psychology and Program in Statistical & Data Sciences Smith College Northampton Massachusetts USA; ^3^ Department of Anthropology University of Massachusetts Amherst Massachusetts USA

**Keywords:** flow‐mediated dilation, hot flashes, perimenopause, physical activity

## Abstract

The menopausal transition is associated with increased risk for cardiovascular disease (CVD). Hot flashes (HF), a cardinal symptom of menopause, have been associated with increased CVD risk, particularly in perimenopausal women. Flow‐mediated dilation (FMD) is an indicator of endothelial function and a subclinical CVD risk factor. Lower FMD has been associated with more HF. As moderate to vigorous physical activity (MVPA) is recognized to reduce CVD risk, our goal was to determine whether higher levels of MVPA change the relationship between HF and FMD in perimenopausal women. Healthy perimenopausal women had HF measured objectively using sternal skin conductance for 24 h. MVPA was determined using 7 days of actigraphy. Endothelial function was measured via brachial artery FMD on the non‐dominant arm. Pearson correlations and multiple regression analyses were used to evaluate relationships between variables. Simple slopes analysis was performed to understand how MVPA moderates the relationship between HF and FMD. Lower FMD tended to correlate with a higher objective HF rate, and this relationship was stronger for HF measured during waking hours. Controlling for age and BMI, HF and the interaction between HF and MVPA were significant predictors of FMD. Simple slope analysis showed a significant HF effect on FMD with lower (−1SD) MVPA, whereas there was no significant relationship between HF and FMD with higher (+1SD) MVPA. These results suggest that MVPA moderates the relationship between FMD and objective HFs in perimenopausal women.

## 1 INTRODUCTION

Perimenopause is the time around the final menstrual period starting when menstrual cycles vary in length ≥7 days up to 12 months of amenorrhea after the final period. In the United States, the average age for the final menstrual period is 52.5 (Gold et al., 2013). Cardiovascular disease (CVD) risk increases dramatically during the perimenopause (El Khoudary et al., 2013; Kannel, 1976; Matthews et al., 2001, 2009, 2017; Samargandy et al., 2020) and may lead to worse outcomes in the postmenopausal years (Matthews et al., 2017). There is a need to evaluate novel and subclinical risk factors in perimenopausal women as a high prevalence of subclinical atherosclerosis has been found in postmenopausal women who are classified as having low‐moderate CVD risk according to conventional factors (Appelman et al., 2015; Lambrinoudaki et al., 2013; Maturana et al., 2015).

Many symptoms of menopause, notably hot flashes (HF), have been associated with long‐term negative health consequences (Davis et al., 2023). Approximately 70% of women experience HF (Gold et al., 2006; Thurston & Joffe, 2011). Hot flashes are a sudden dissipation of heat that may be accompanied by cutaneous vasodilation and sweating. Hot flashes are caused by reductions in estradiol that lead to increased activity of kisspeptin/neurokinin B/dynorphin (KNDy) neurons in the preoptic area of the hypothalamus (Mittelman‐Smith et al., 2015; Rance et al., 2013). The increased KNDy neuron activity triggers cutaneous vascular and sweat gland effectors causing a HF.

The frequency and severity of HF are associated with CVD risk factors and subclinical CVD risk (Bechlioulis et al., 2010; Gast et al., 2008, 2011; Herber‐Gast et al., 2015; Thurston et al., 2008, 2021; Thurston, Chang, et al., 2017; Thurston & Joffe, 2011). For example, women with HF had higher total cholesterol, blood pressure, and body mass index (BMI) compared with women who did not report HF (Gast et al., 2008). Endothelial dysfunction is a hallmark subclinical condition that precedes atherosclerosis (Zeiher et al., 1991a, 1991b) and is prognostic of future CVD events and mortality (Gokce et al., 2003; Matsuzawa et al., 2015; Yeboah et al., 2007, 2009). Flow‐mediated dilation (FMD) is a measure of endothelial function that is predictive of future CVD events and subclinical CVD (Shimbo et al., 2007; Yeboah et al., 2009). Endothelial function has been shown to decrease in women around the transition to menopause (Moreau, Hildreth, et al., 2012; Witkowski & Serviente, 2018); however, in these studies, HF experience has not been considered.

Endothelial dysfunction is related to greater subjectively reported HF severity in early menopausal women (Bechlioulis et al., 2010). Thurston et al. (Thurston et al., 2008) reported that women aged 42–52 who reported HF had lower FMD compared with women who did not report HF. In a study relating self‐reported HF with FMD, Bechlioulis et al. (Bechlioulis et al., 2010) found that the severity of HF in early postmenopausal women (aged 42–55) was strongly associated with FMD where 75% of the variance in FMD was predicted by HF severity. The use of objective hot flash measurement via sternal skin conductance has strengthened and extended the evidence supporting a relationship between HF and FMD. Recently, using objectively measured HF, Thurston et al. (Thurston, Chang, et al., 2017) reported that, in women aged 40–53, that is, women around the age of the final menstrual period, the presence and increased frequency of measured HF were significantly related to lower FMD.

One factor not addressed in the studies relating HF to endothelial function and CVD risk is physical activity. Habitual moderate to vigorous physical activity (MVPA) is recognized as an effective lifestyle behavior to reduce CVD risk although less is known about the effects of exercise on endothelial function during the menopausal transition as studies have focused on postmenopausal women. A randomized controlled trial demonstrated that while exercise training improved endothelial function in premenopausal women and midlife men, there was no change in postmenopausal women with aerobic exercise training (Pierce et al., 2011). Cross‐sectional comparisons of aerobically exercise‐trained and sedentary postmenopausal women also do not show a difference in FMD (Pierce et al., 2011; Santos‐Parker et al., 2017). Training studies incorporating other exercise types such as high‐intensity interval training and strength training have shown that regardless of training intensity or type, exercise training in postmenopausal women did not result in improved FMD (Casey et al., 2007; Klonizakis et al., 2014). However, data from our lab show that in response to an acute bout of moderate treadmill exercise, FMD increased in peri‐ but not postmenopausal women (Serviente et al., 2019). Therefore, the efficacy of MVPA to affect FMD in perimenopausal women requires further investigation.

Physical activity can influence HF experience in midlife women. Using objective measures of HF, our recent studies have shown that habitual physical activity and sedentary behavior can influence the HF experience, (Witkowski, White, Shreyer, Brown, & Sievert, 2024) and HF can be preceded by acute increases in physical activity (Witkowski, White, Shreyer, Garcia, et al., 2024). Given that more HF have been associated with reduced FMD in perimenopausal women (Thurston, Chang, et al., 2017), the goal of this study was to determine whether habitual physical activity changes the association between objectively measured HF and FMD in perimenopausal women.

## 2 MATERIALS AND METHODS

The Smith College Institutional Review Board approved the protocol (IRB ID: 18–108), and participants provided written informed consent before beginning any study procedures.

### 2.1 Screening

Participants were healthy perimenopausal women aged 43–54 years. Perimenopause was defined based on Stages of Reproductive Aging Workshop (STRAW+10) guidelines (Harlow et al., 2012) which includes people with changes in menstrual cycle length of ≥7 days than normal, up to less than 1 year of amenorrhea. Most participants identified as non‐Hispanic and of European ancestry. Participants were ineligible if they had a history of CVD, chronic menstrual irregularities (i.e., polycystic ovary syndrome), surgical menopause, if they were pregnant or lactating, if they had used menopausal symptom treatment, hormone therapy, or oral contraceptives in the past 6 months, or if they were current smokers or had smoked in the last 6 months.

Participants had a fasting blood sample drawn and were screened for levels of total cholesterol (TC), triglycerides (TG), low‐density lipoprotein cholesterol (LDL‐C), high‐density lipoprotein cholesterol (HDL‐C), and fasting plasma glucose. Participants were excluded from the study if fasting plasma glucose was >126 mg/dL, or if fasting plasma lipids were at any of the following levels: LDL‐C >160 mg/dL, HDL‐C <40 mg/dL, TG >200 mg/dL, or TC >240 mg/dL. Height, weight, waist‐to‐hip ratio, and resting blood pressure (Muntner et al., 2019) were measured on all participants, and participants were excluded from the study if resting blood pressure was >140/>90 mmHg or if body mass index was <18.5 or >35 kg/m^2^.

Of 208 people screened, 77 eligible people enrolled in the study. Data from participants with incomplete data (i.e., missing FMD or HF data), changes in menstrual cycle or medication, or those who voluntarily withdrew were not included. Eventually, 59 participants were included in this analysis.

### 2.2 Brachial flow mediated dilation

Brachial artery FMD was measured similarly to the previously published protocol in the lab (Serviente et al., 2016, 2019) which adheres to the expert consensus guidelines for flow‐mediated dilation (Greyling et al., 2016a, 2016b; Thijssen et al., 2019). For participants who experienced menstrual periods, FMD was assessed on days 2–5 of the menstrual cycle. Controlled conditions included a familiarization trial that was performed for all participants during the screening visit. FMD was assessed in the early morning in all participants to control for diurnal variation. Participants were instructed to fast for at least 6 h, to avoid alcohol, caffeine, exercise, and smoking for 12 h, and to refrain from pain medication and phosphodiesterase inhibitors for 24 h, and to stop taking any vitamins and supplements 72 h before the assessment. All measurements took place in a quiet, temperature‐controlled room with participants in the supine position for at least 25 min prior to data collection. Measurements were obtained from a non‐dominant brachial artery longitudinally proximal to the cubital fossa using duplex ultrasound (Terason 3300 Ultrasound System, Terason, Ormond Beach, FL, USA) with a 60° insonation angle to image the artery and blood flow. The probe was affixed to a stereotactic clamp to maintain the ultrasound head position on the arm. A rapid inflation cuff (D. E. Hokanson, Bellevue, WA) was placed around the largest part of the forearm distal to the probe. The diameter of the brachial artery and the velocity of blood flow were continuously captured using FMD Studio software (version 4.0, FMD Studio, Quipu, Pisa, Italy, RRID:SCR_018325). The measurement protocol was 11 min in total including 2 min of rest, 5 min of forearm cuff occlusion (200 mmHg), and 4 min of reactive hyperemic response post‐cuff release. Blood pressure and heart rate were measured each minute on the dominant arm using an automatic blood pressure cuff with a 3‐lead electrocardiogram to verify a stable heart rate and blood pressure.

Data were processed using the FMD Studio software and evaluated to obtain average baseline brachial artery diameter, average peak artery diameter, shear rate area, and time to peak diameter. The percent change in FMD (FMD%) was calculated as the percentage of the difference between the two artery diameters normalized to the baseline diameter: [(peak diameter – baseline diameter/baseline diameter) *100]. Shear rate was defined as (4*mean blood velocity)/internal diameter. Time to peak was calculated as the time in seconds from the release of the forearm cuff to the time of the peak diameter reading.

### 2.3 Hot flash assessment

Objective HF was measured for 24 h using an ambulatory monitor for continuous measurement of sternal skin conductance (Biolog monitor, UFI, Morro Bay, CA, USA) (Sievert, 2013). Skin conductance was recorded using silver/silver chloride electrodes placed 4 inches apart across the mid‐sternum with a 0.5 constant voltage circuit. The monitor had a button that participants were instructed to push when they felt a HF to mark subjective HF. Participants were also provided with a paper log where they reported time‐in‐bed, time‐out‐of‐bed and could report a subjective HF retrospectively if pressing the button was not possible at the time.

The monitor recordings were analyzed using the 3991x GPP Biolog DPS software (version 1.2) and FlashTrax software (version 2.1) (UFI, Morro Bay, CA, USA). Hot flashes were coded following the established criteria of an increase in 2 μ℧ over 30 s, and a 20‐min post‐HF lockout was applied. The universality of the 2 μ℧ criteria has been questioned (Thurston et al., 2009) and there can be a great amount of variation in the amplitude of the output from the monitors. Some recordings show the characteristic shape of a hot flash, but do not quite reach the 2 μ℧ criteria. For example, studies have used 1.78 and 1.2 μ℧ criteria (Hanisch et al., 2007; Savard et al., 2014). In our study, in order to determine whether these hot flashes should be counted, we relied on confirmation with a subjective report. If objective HF did not meet the established criteria, although the characteristic change in sweating pattern was observed, an objective HF was recorded if it was accompanied by the participant's subjective report. Hot flashes that did not meet the 2 μ℧ criteria were not counted if there was no subjective report. This methodology has been used in multiple studies (Thurston et al., 2009; Witkowski, White, Shreyer, Brown, & Sievert, 2024; Witkowski, White, Shreyer, Garcia, et al., 2024) and is acceptable in the field (Fisher & Thurston, 2016; Sievert, 2013). Participants were instructed not to engage in exercise during the monitoring period but could engage in physical activity of daily living. Only records with at least 9‐h participant wear time were included.

Hot flash variables assessed in this analysis include daytime HF, nighttime HF, and total HF. Total HF was the total number of HF experienced during total wear time. Hot flash rate was calculated as the HF count divided by monitor wear time. Nighttime HF count was the number of HF during time‐in‐bed from participant's bedtime log. The remaining time was defined as daytime, and HF during these hours were counted as daytime HF. The night wear time and day wear time were used to calculate night HF rate and day HF rate, respectively.

### 2.4 Habitual physical activity

Self‐reported habitual physical activity levels were determined by the International Physical Activity Questionnaire (IPAQ). Objective physical activity was measured using the GT3X ActiGraph accelerometer (wGT3XP‐BT: ActiGraph, Pensacola, FL, USA, RRID:SCR_008399) worn on the non‐dominant wrist for 7 consecutive days with the exception of removal during water‐related activities (e.g., swimming, showering). Habitual Physical activity was assessed from 7‐day actigraphy that occurred before the HF monitoring, not when the HF monitor was worn. Monitors were programmed to measure 1‐min epochs.

The raw acceleration signal output was downloaded using the ActiLife software (version 6.8.1; ActiGraph, Pensacola, FL, USA). A valid day of wear was considered 21.6 h to provide sufficient reliability (>90%) for all analyses (Riddoch et al., 2007) regardless of a weekend day inclusion. The GGIR R package (version 2.9–0) was used to obtain the objective physical intensity in average minutes per day (Migueles et al., 2019; Van Hees et al., 2013, 2015). Moderate activity was the average minutes of moderate physical activity (e.g., carrying light loads) per valid day. Vigorous activity was the average minutes of vigorous physical activity (e.g., carrying heavy loads, aerobics) per valid day. Objective moderate‐vigorous activity (MVPA) is the sum of the average minutes of moderate and vigorous physical activity per valid day. Following the published cut‐point recommendations of Hildebrand et al. (Hildebrand et al., 2014, 2017) for adults aged 21–61, the threshold for cut points for light activity was set as 44.8, moderate activity as 100.6, and vigorous activity as 428.8.

### 2.5 Blood hormone analysis

Serum blood samples were collected and used to assess 17β estradiol (E2), and follicle‐stimulating hormone (FSH). The blood collection was completed on the same day as FMD assessment, and the same restrictions were followed as described above. Collected venous whole blood was gently, continuously mixed, and processed to separate plasma and serum. They were aliquoted and stored at −80°C until analysis.

The quantification of FSH was performed using a commercially available ELISA kit (RayBiotech, Peachtree Corners, GA, USA (ELH‐FSH‐1); https://www.raybiotech.com/human‐fsh‐elisa‐elh‐fsh). The serum samples and kit components were thawed to room temperature before use. All items in the procedure except for samples, controls, MilliQ water, plate washer, and plate shaker were provided by the kit. All samples were run in triplicates, and the standards were run in duplicate, following the manufacturer's protocol. Briefly, standards were made from a 25 ng/mL stock solution that was serially diluted to produce a standard curve of 2000 pg/mL to 8.19 pg/mL. The lower level of detection is 8 pg/mL (0.11 mIU/mL), according to the manufacturer. The 1x assay diluent was used as a zero standard. The serum from each participant was diluted 4‐fold using 1x assay diluent and mixed well by pipetting and a brief centrifugation before loading on the 96‐well plate. The samples, standards, and controls were added to the plate and incubated for 2.5 h at room temperature. Next, samples were washed and prepared; biotin antibodies were added to each well and incubated for an hour. Next, the plate was washed then incubated with prepared streptavidin solution for 45 min. After a final washing, the TMB One‐Step substrate reagent was added and incubated for 30 min in a dark room at room temperature without shaking. The reaction was stopped using a stop solution, and the raw absorbance values were obtained at 450 nm immediately. A 4‐parameter logistics standard curve was fitted (*R* ^2^ = 0.99), and the concentrations for the samples were calculated using the curve. If the CV% for the triplicates was higher than 10%, the replicate that was ±2 standard deviations from the mean of the other two replicates was excluded from the average concentration calculation. The average coefficient of variation for samples was 4.67% ± 2.60. The conversion to International Unit (mIU/mL) from (pg/mL) was calculated using 1 mIU/mL = 70 pg/mL, per manufacturer's recommendation.

The quantification of 17β estradiol (E2) was performed using the commercially available ELISA kit (AbCam, Waltham, MA, USA; https://www.abcam.com/products/elisa/17‐beta‐estradiol‐elisa‐kit‐ab108667.html). The serum samples (stored at −80°C) were thawed to room temperature directly prior to analysis. Per manufacturer's recommendation, the samples were mixed gently for 5 min before testing. All items in the procedure except for samples, controls, MilliQ water, plate washer, and incubator were provided by the kit. All samples were run in triplicates, and the standards were run in duplicate. The standards were placed on the 96‐well plate to prepare for a standard curve of 20 pg/mL to 2000 pg/mL. The lower level of detection is 8.68 pg/mL, according to the manufacturer. The samples and controls were also placed in their respective wells, and 17‐ꞵ estradiol‐HRP conjugate was added to each well. A blank well was used as a substrate blank. The wells were covered and incubated for 2 h at 37°C. The plate was washed three times with the wash buffer. The TMB substrate solution was added into all wells and incubated for 30 min at room temperature in the dark before adding a stop solution. The raw absorbance values were obtained at 450 nm immediately. A 4‐parameter logistics standard curve was fitted (*R* ^2^ = 0.99), and the concentrations for the samples were calculated using the curve. For sample concentrations below the minimum level of detection, the minimum level of detection was imputed. If the CV% for the triplicates was higher than 10%, the replicate that was ±2 standard deviations from the mean of the other two replicates was excluded from the average concentration calculation. The average coefficient of variation for samples was 4.9% ± 3.2.

### 2.6 Data analysis

All statistical analyses were performed using R (version 4.2.3, R Foundation for Statistical Computing, Vienna, Austria) and RStudio software (2023.06.2 + 561, Posit Software, PBC, Boston, MA).

The means, standard deviations, and distributions of participant variables were calculated and assessed. Pearson correlations were used to assess the correlations between FMD and objective HF rate (i.e., total, daytime, and nighttime HF), FMD and habitual MVPA, and total objective HF rate and MVPA. Since HF data was not normally distributed, correlations between HF and FMD were also evaluated using the nonparametric Spearman's rank correlation coefficient (ρ). A Welch's two‐sample *t*‐test allowing for unequal variances was conducted to determine if FMD was different between participants who experienced objective HF versus those who did not. All data were evaluated for adherence to model assumptions for each statistical test.

Multiple linear regression analyses were performed to determine the relationships between objective hot flash rate, objective moderate‐vigorous physical activity (MVPA), and flow‐mediated dilation (FMD), including an interaction model to address whether MVPA changes the relationship between objective HF rate and FMD. Additional predictors known to influence FMD, including age, BMI, and cardiovascular health parameters (e.g., blood pressure, cholesterol, fasting glucose), were also considered in modeling. When control variables had multiple measures (e.g., SBP and DBP for BP), the measure with the highest correlation with FMD was chosen for inclusion in the model while the others were excluded to avoid collinearity. Models were constructed to predict FMD in the following manner: (1) multiple linear regression with objective HF rate and objective MVPA only (additive model), (2) multiple linear regression with objective HF rate and MVPA (interaction model), (3) model 2 predictors plus age and BMI, (4) model 3 predictors plus E2 and FSH, (5) model 4 predictors plus CVD variables of TC, FPG, and SBP. For the interaction models, when the interaction term (Objective HF × MVPA) was significant (*p* < 0.05), simple slope analyses were conducted to estimate the effects of the independent variable HF on FMD for higher MVPA level (1 standard deviation (SD) above the MVPA grand mean) and lower MVPA level (1 SD below the MVPA grand mean). The ꞵ‐coefficient, *p*‐value, total variance (*R* ^2^), and adjusted *R* ^2^ were reported for each of the five models. Residual diagnostics were performed for the chosen models to evaluate the model assumptions.

## 3 RESULTS

The participant characteristics are reported in Table 1. The study included 59 perimenopausal participants who were healthy, physically active, and free of CVD risk factors. Blood pressure, cholesterol, BMI, and fasting plasma glucose of the participants were within normal range for a general, healthy population. Both E2 and FSH levels varied for our participant group as expected for perimenopausal women. The average objective MVPA was 87 ± 37 min/day which was higher than recommended PA levels for this age group from the Physical Activity Guideline for Americans (at least 150–300 min/week of moderate or 75–150 min/week of vigorous PA or an equivalent combination of moderate‐vigorous intensity aerobic activity), for this age group (Piercy et al., 2018).

**TABLE 1:** Participant Characteristics.

Characteristic	Mean ± SD
Age (year)	49 ± 3
Height (cm)	164 ± 6
Weight (lb)	147 ± 28
BMI (kg/m^2^)	24.7 ± 4.9
Waist: Hip Ratio	0.77 ± 0.07
HDL (mg/dL)	69 ± 14^b^
LDL (mg/dL)	106 ± 25^f^
TRG (mg/dL)	79 ± 33^f^
TC (mg/dL)	185 ± 30^a^
FPG (mg/dL)	88 ± 9^a^
SBP (mmHg)	110 ± 12
DBP (mmHg)	70 ± 7
E2 (pg/mL)	33.1 ± 32.8^b^
FSH (mIU/mL)	8 ± 6^b^
Objective HF count (count/wear day)	5 ± 7^c^
Objective HF rate (count/wear hour)	0.21 ± 0.27^c^
Objective daytime HF count (count/wear day)	3 ± 5^d^
Objective daytime HF rate (count/wear hour)	0.21 ± 0.30^d^
Objective nighttime HF count (count/wear day)	2 ± 2^e^
Objective nighttime HF rate (count/wear hour)	0.22 ± 0.32^e^
Total HF monitor weartime (hour)	19 ± 10^a^
Subjective PA (MET‐min/wk)	3269 ± 2625
Objective MVPA (min/day)	87 ± 37
FMD (%)	4.82 ± 3.22
Baseline diameter (mm)	3.31 ± 0.37
Maximum diameter (mm)	3.47 ± 0.39
Shear rate AUC (s^−1^)	48,627 ± 48,761
TTP (s)	50 ± 23

*Note*: *n* = 59, unless otherwise noted.

Abbreviations: AUC, area under curve; BMI, Body Mass Index; DBP, diastolic blood pressure; E2, 17β‐estradiol; FMD, flow‐mediated dilation; FPG, fasting plasma glucose; FSH, follicle‐stimulating hormone; HDL, high‐density lipoprotein cholesterol; HF, hot flash; LDL, low‐density lipoprotein cholesterol; MVPA, moderate‐vigorous physical activity; PA, physical activity; SBP, systolic blood pressure; TC, total cholesterol; TRG, triglycerides; TTP, time to peak.

^a^ *n* = 58.

^b^ *n* = 57.

^c^ *n* = 52.

^d^ *n* = 50.

^e^ *n* = 48.

^f^ *n* = 41.

There were 52 participants with valid HF objective recordings and 37 of these participants experienced objective hot flashes during the monitoring period while 15 did not. Participants had an average of 5 ± 7 recorded hot flashes per monitoring session (0.21 ± 0.27 HF/h). Flow‐mediated dilation was not different between participants who experienced HF (4.26% ± 2.81) versus those who did not (5.29% ± 3.23, *t*(23.03) = −1.08, *p* = 0.29).

**FIGURE 1:**
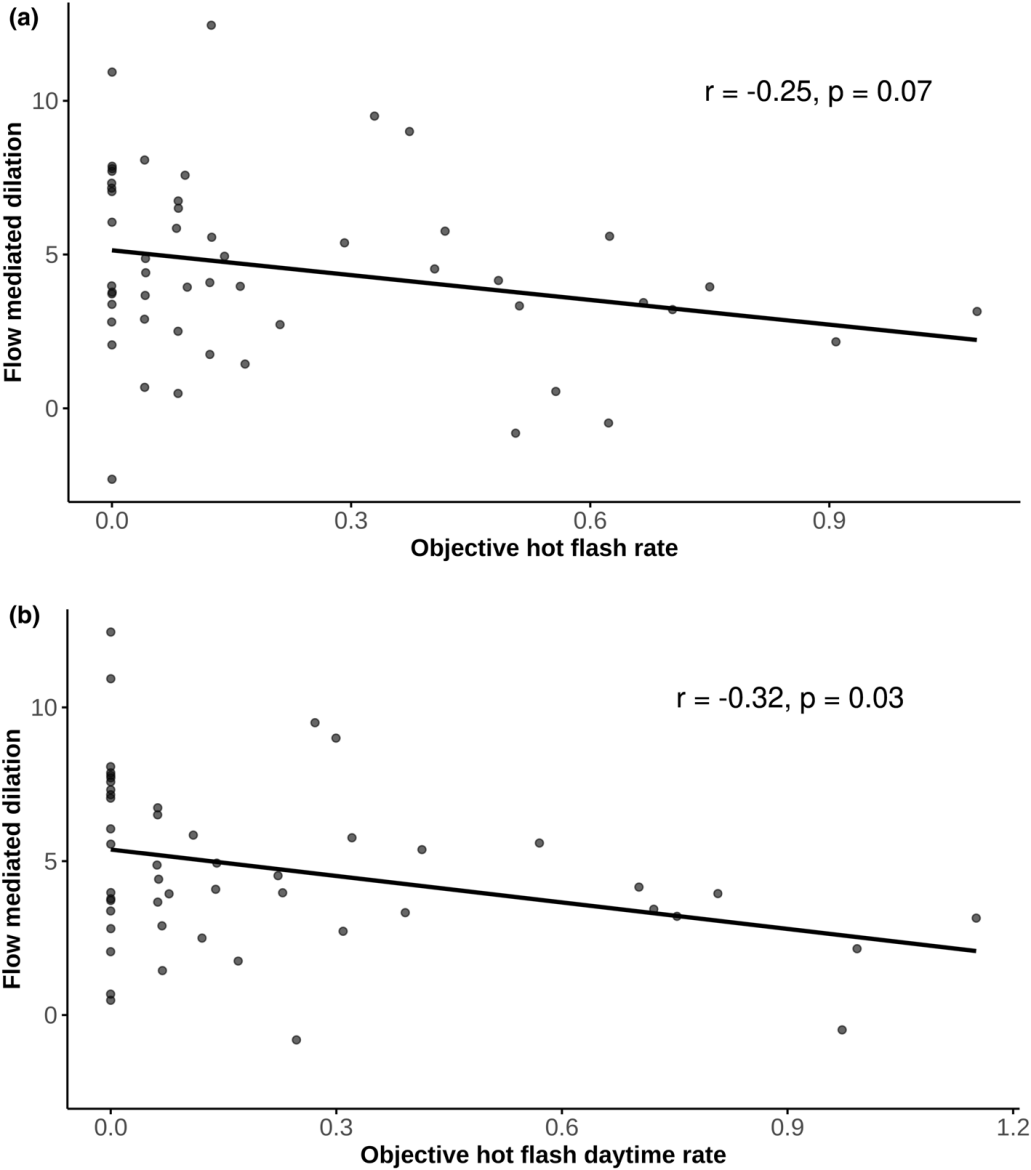
Flow mediated dilation and hot flash relationships. (a) Total hot flash rate, (b) Daytime only hot flash rate.

Lower FMD (%) tended to be correlated with a higher objective HF rate (*r* (52) = −0.25, *p* = 0.07) (Figure 1a). The correlation was stronger and significant for HF measured during waking hours (*r* (48) = −0.32, *p* = 0.03) (Figure 1b) but not those measured while the participant was in bed (*r* (46) = −0.15, *p* = 0.31). Correlation results evaluated with nonparametric Spearman rank correlation tests yielded similar results (total objective HF, *ρ* (52) = −0.25, *p* = 0.07; waking HF, *ρ* (48) = −0.32, *p* = 0.02).

The correlation between FMD and objectively measured MVPA was not significant (*r* = −0.15, *p* = 0.31). Results from multiple regression modeling are found in Table 2. Objectively measured levels of MVPA modified the association between FMD and objective HF rate. In the model controlling for age and BMI (model 3), HF (ꞵ[SE] = −11.10[4.24], *p* = 0.01) and the interaction between HF and MVPA (ꞵ[SE] = 0.09[0.04], *p* = 0.04) were significant predictors of FMD (adj *R* ^2^ = 0.13, *F* (5, 46) =2.54, *p* = 0.04). BMI was also a significant predictor in this model (ꞵ[SE] = 0.17[0.08], *p* = 0.03). The interaction was such that higher levels of MVPA were related to a less strongly negative relationship between objective HF rate and FMD.

**TABLE 2:** Regression models predicting flow mediated dilation.

Variables	FMD				
	Model 1 (ns)	Model 2 (*)	Model 3 (**)	Model 4 (*)	Model 5 (ns)
HF	−2.83*	−9.47**	−11.10**	−10.60**	−11.00**
MVPA	−0.008	−0.02	−0.02	−0.02	−0.02
HF × MVPA		0.07	0.09**	0.09*	0.09*
Age			0.08	0.04	−0.01
BMI			0.17**	0.17*	0.15
E2				−0.01	−0.012
FSH				0.03	0.01
TC					−0.002
FPG					−0.006
SBP					0.04
*R* ^2^	0.07	0.12	0.22	0.23	0.25
Adj *R* ^2^	0.04	0.07	0.13	0.11	0.06

*p < 0.1; **p ≤ 0.05.

Abbreviation: ns, not significant.

**FIGURE 2:**
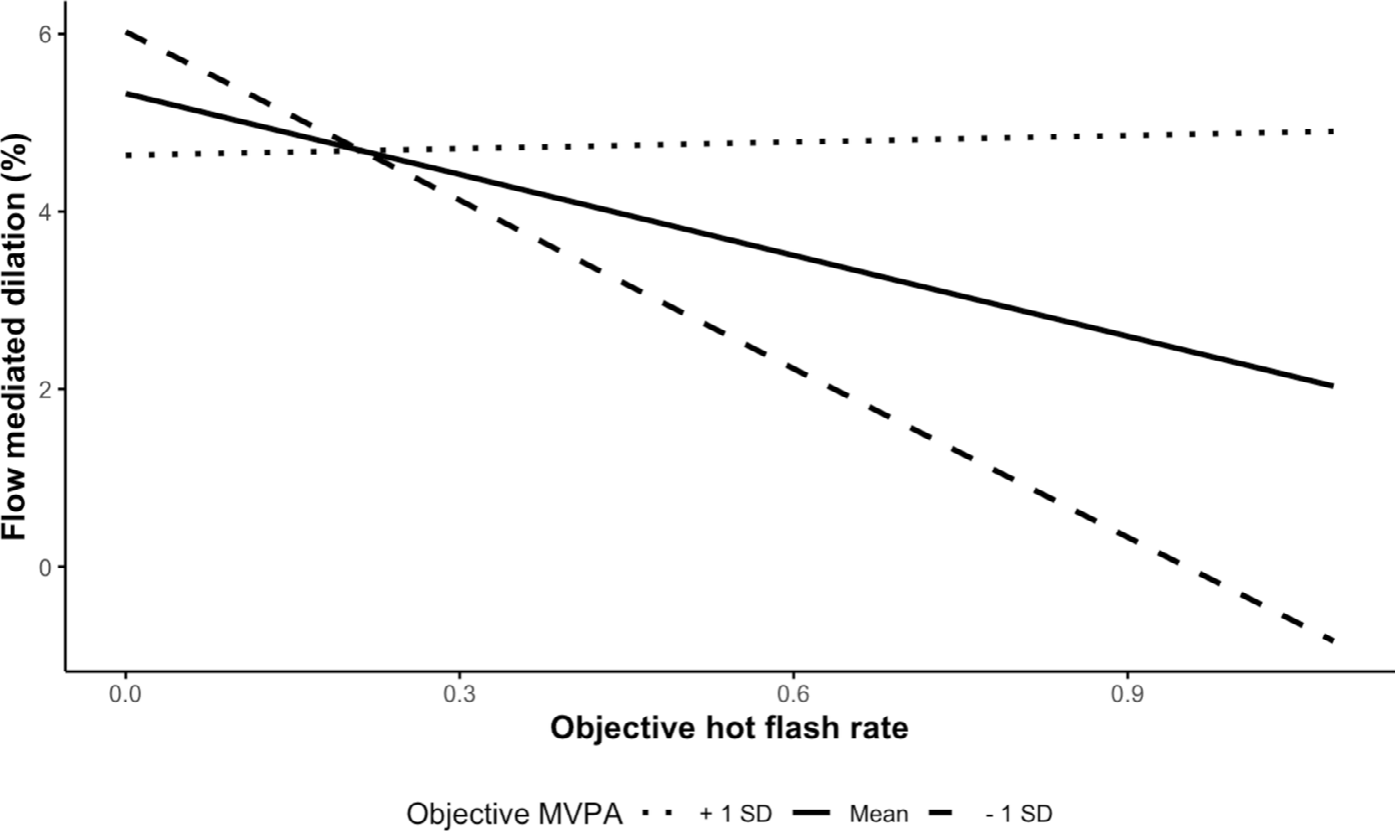
Differences in the flow mediated dilation and hot flash relationship by higher and lower moderate to vigorous physical activity.

The simple slopes analysis of the interaction effect between HF and MVPA found that the effect of HF rate on FMD at lower MVPA (one SD below the mean) was significantly negative (ꞵ[SE] = −6.5[2.3], *p* = 0.007), whereas the HF rate effect on FMD at higher MVPA (one SD above the mean) was not significant (ꞵ[SE] = 0.19[2.0], *p* = 0.90) indicating that for higher levels of MVPA changed the significant relationship between objective HF and FMD (Figure 2). We also note that the HF rate effect on FMD when MVPA was at the sample mean was also significant and negative, (ꞵ[SE] = −3.2[1.4], *p* = 0.03).

To evaluate whether CVD risk factors and hormones including estradiol and/or FSH played a role in the relationship between HF and FMD, we considered these factors as covariates in our models (Models 4 and 5) but they did not explain a significant portion of the variance in FMD in either model (Table 2).

## 4 DISCUSSION

FMD is considered a subclinical CVD risk factor, and low FMD has been associated with future CVD events (Thijssen et al., 2011; Yeboah et al., 2007, 2009). Accumulating evidence suggests that women who experience HF prior to menopause, including perimenopausal women, have increased risk for CVD. As regular MVPA is known to reduce CVD risk, we aimed to understand whether MVPA changed the association between HF and CVD risk in perimenopausal women. Our results support prior findings that more objectively measured HF, particularly those occurring in waking hours, were associated with lower FMD, an indicator of endothelial dysfunction. Importantly, we report for the first time that when taking into account objectively measured physical activity, MVPA moderates the relationship between HF and FMD. These findings extend the limited available data on the benefits of more MVPA for those with HFs and on CVD risk around the transition to menopause.

This study focused exclusively on women in the perimenopausal stage of menopause, when menstrual cycles are variable but women have not yet reached 12 months beyond their final menstrual period. Brachial artery FMD declines across the stages of menopause independent of aging (Moreau, Hildreth, et al., 2012) and Moreau et al. showed that the greatest decline in FMD occurred during the perimenopausal stage. While they did note a trend to a negative association between self‐reported HF frequency across all participants (*r* = −0.17, *p* = 0.08), in that study they did not consider objectively measured hot flashes. The propensity of the evidence shows that the relationship between FMD and HF is evident during the early years of the menopause transition (Bechlioulis et al., 2010; Hildreth et al., 2018; Thurston et al., 2008; Thurston, Chang, et al., 2017). Notably, using objective measures of HF, Thurston et al. (Thurston, Chang, et al., 2017) reported that the presence of HF and frequency of HF were associated with lower FMD among the younger tertile of women in their sample (age 40–53 years), but not in older groups of women. They also reported that the association between HF and FMD was strongest in perimenopausal women. In that study, physical activity was assessed using self‐report via the International Physical Activity Questionnaire (IPAQ) and used as a covariate in modeling. In the current study, MVPA was assessed objectively over 7 days, and objectively measured HF were a significant predictor of FMD in modeling that accounted for the interaction between MVPA and HFs (i.e., when MVPA was at the sample mean). This is notable because participants were healthy with no CVD risk and relatively active with 87 ± 37 min of MVPA/day, which complies with Physical Activity Guidelines for MVPA (U.S. Department of Health and Human Services, 2018).

Studies using subjectively assessed vasomotor symptoms (VMS), which include both HF and night sweats more broadly, suggests that early VMS may have associations with clinical CVD outcomes later in life. In the Women's Ischemia Syndrome Evaluation (WISE) study, women reporting onset of VMS before the age of 42, had lower FMD and higher CVD mortality in the subsequent 6 years (Thurston, Johnson, et al., 2017). Data from the Study of Women Across the Nation (SWAN) study (Thurston et al., 2021) showed in women aged 42–52 at baseline and followed over 22 years that participants with frequent VMS (≥6 days) at baseline, had a 50% greater risk of CVD events compared with no VMS (hazard ratio [HR] [95% CI], 1.51 [1.05–2.17], *p* = 0.03). A systematic review and meta‐analysis showed that in women under the age of 60, there was a significant relationship between VMS and cardiovascular events, but not in women over 60 (Armeni et al., 2023).

For many, the experience of hot flashes changes over time. Data from SWAN wherein participants were queried on their VMS over the years around the menopause transition showed that those who had early onset VMS, up to 10 years before their final menstrual period, had the highest carotid intima media thickness (IMT), comparable to more than 4 years of aging (Thurston et al., 2016). However, a recent pooled analysis demonstrated that both early and late VMS onset was associated with future CVD events (Zhu et al., 2020). Overall, most evidence suggests that hot flashes prior to and around the final menstrual period are associated with subclinical CVD risk and clinical CVD.

Physical activity is recognized for its benefits to cardiovascular health. However, the effect of greater amounts of MVPA on FMD in perimenopausal women remains equivocal. The majority of studies that have addressed the effect of physical activity and exercise have exclusively evaluated postmenopausal women, who have had at least 1 year of amenorrhea. In these studies, the effect of an exercise training intervention to improve FMD is inconsistent, and cross‐sectional studies similarly fail to consistently show differences in physically active versus lower active women (Lew et al., 2022; Moreau et al., 2024). Moreau et al. (Moreau, Hildreth, et al., 2012) showed a significant association between VO_2peak_ and brachial artery FMD in participants including all menstrual cycle stages (*r* = 0.35, *p* < 0.001). Data from our lab are some of the only data including perimenopausal women and show a reduction in FMD through the stages of menopause, even in highly active pre‐, peri‐, and postmenopausal women (Serviente & Witkowski, 2019). However, we have also shown that there is an increase in FMD in response to acute exercise in perimenopausal women (Serviente et al., 2016) particularly in those with lower cardiorespiratory fitness who do not meet Physical Activity Guidelines (Physical Activity Guidelines Advisory Committee, 2008; Serviente et al., 2016). Data from the current study showed no role for MVPA to predict FMD with or without controlling for CVD risk factors, potentially due to all participants being relatively active (Tucker et al., 2011).

Regarding the effect of physical activity on HF, there are mixed results on whether physical activity changes the experience of HF (Witkowski et al., 2023) and this may be due in part to whether subjective or objective measures of HF and physical activity are employed. Recent evidence from our lab showed that greater self‐reported vigorous physical activity was associated with increased subjectively reported but not objectively measured HF during waking hours (Witkowski, White, Shreyer, Brown, & Sievert, 2024). In that analysis, we also found that increasing sedentary time by 1 h increased objectively measured hot flashes by 7% during nighttime (in bed) hours. In another analysis, we found that a 1 standard deviation increase in actigraphy‐assessed physical activity during the day was associated with a 31% increase in the odds of an objectively measured HF (Witkowski, White, Shreyer, Garcia, et al., 2024), suggesting as others have (Elavsky et al., 2012; Freedman & Krell, 1999; Gibson et al., 2014; Thurston et al., 2005), that acute changes in physical activity can be a trigger for HF but not all increases in PA lead to HF. It remains unclear whether this is the case in women with higher levels of habitual physical activity. Even though the effect of physical activity on HF remains unclear, according to the current study, higher amounts of MVPA can moderate the relationship between HF and the subclinical CVD risk factor, FMD in perimenopausal women.

The mechanisms underlying the association between HF and FMD and the capacity of MVPA to alter that relationship are unclear. Declines in estradiol are attributed to changes in the hypothalamus (Mittelman‐Smith et al., 2015; Rance et al., 2013) and thermoregulation (Freedman, 2001) that underlie hot flashes. Estradiol and exercise are both known to influence the production of the vasodilator nitric oxide (NO) via genomic and non‐genomic pathways (Tamariz‐Ellemann et al., 2023). Estradiol influences endothelial NO synthase (eNOS) through the ERɑ and G‐protein‐coupled receptors (Chambliss & Shaul, 2002; Fredette et al., 2018) and exercise increases eNOS protein and ERRɑ expression in early postmenopausal women (Nyberg et al., 2017). In postmenopausal women, estrogen treatment and supplementation of tetrahydrobiopterin (BH4), an important cofactor for eNOS activity, increased FMD (Moreau, Meditz, et al., 2012) and was necessary to increase FMD with 12 weeks of exercise training (Moreau et al., 2013). Furthermore, inhibition of eNOS using L‐NAME reduced cutaneous vasodilation during a hot flash (Hubing et al., 2010), indicating that eNOS and NO contribute to vasodilation during a HF. While there appear to be common pathways through estradiol and eNOS to the endothelium with FMD, HF, and physical activity, these relationships with estradiol are not consistent. There is accumulating evidence, including some from our lab, showing that there are stronger associations between FSH and FMD (Hildreth et al., 2018; Serviente & Witkowski, 2019) compared with estradiol. However, our data presented herein do not support any role for either estradiol or FSH. Alternative explanations to the effect of MVPA to reduce the relationship between HF and FMD may include the ability of exercise to reduce inflammation (Nørregaard et al., 2023; Ryan et al., 2014) and/or autonomic nervous system activation related to hot flashes (Joyner & Green, 2009; Sánchez‐Delgado et al., 2023). More studies are needed to better understand not only the mechanisms relating HF to endothelial dysfunction in perimenopausal women, but also those that underlie the ways regular exercise moderates this relationship.

### 4.1 Limitations

While the strengths of this study include the focus on perimenopausal women and objective measures of both HF and physical activity, there are notable limitations. First, this study design only improves our knowledge regarding associations between measures rather than mechanisms. Second, this study is limited by a small sample size of women who are healthy and physically active. The study by Thurston et al., showing similar relationships between HF and FMD included 44 perimenopausal women (Thurston, Chang, et al., 2017). Therefore, these findings will need to be confirmed in a larger sample with a greater range of physical activity behavior and health characteristics to see if more MVPA has the same effect on the relationship in a broader sample. Further, although we found no contribution of estradiol in any model, we measured estradiol via immunoassay, which may not have the sensitivity to detect estradiol levels in the lowest range (Rosner et al., 2013). For studies concerning menopause, it is recommended to use a sensitive technique such as LC/MS (Pinkerton et al., 2020). Finally, HF burden is known to vary according to race and ethnicity, with Black women reporting more VMS and more severe VMS compared with other races and ethnicities (Gold et al., 2006; Kochersberger et al., 2024). The sample in this study was majority white; therefore, future studies should address these relationships in a more diverse sample of women.

## 5 CONCLUSIONS

These data support a role for greater amounts of moderate to vigorous physical activity to reduce the negative association between hot flashes and endothelial dysfunction in perimenopausal women.

## AUTHOR CONTRIBUTIONS

SW and LLS conceived and designed the research. SW, TT, LM, SB, and JB performed the experiments. TT, SB, JB, and SW analyzed the data. SW, and TT drafted the manuscript. SW, TT, and RG interpreted results of experiments. TT prepared figures. SW, TT, LLS, and RG edited and revised the manuscript. SW, TT, LM, SB JB, RG, and LLS approved the final version of the manuscript.

## FUNDING INFORMATION

This research was supported by NIH (NHLBI) 1R15HL145650‐01A1 (Witkowski), Smith College McKinley Fellowship (Tha Ra Wun), and the Smith College STRIDE Fellowship (Buszkiewicz).

## CONFLICT OF INTEREST STATEMENT

Authors have no conflicts of interest.

## ETHICS STATEMENT

This study was conducted in compliance with the ethical standards set forth by the Institutional Review Board at Smith College (Approval Number: 18‐108). All participants provided written informed consent before beginning any study procedures.

## Data Availability

The data that support the findings of this study are available from the corresponding author upon reasonable request.
